# Mitochondrial Calcium Uptake Is Instrumental to Alternative Macrophage Polarization and Phagocytic Activity

**DOI:** 10.3390/ijms20194966

**Published:** 2019-10-08

**Authors:** Serena Tedesco, Valentina Scattolini, Mattia Albiero, Mario Bortolozzi, Angelo Avogaro, Andrea Cignarella, Gian Paolo Fadini

**Affiliations:** 1 Department of Medicine, University of Padova, Via Giustiniani 2, 35128 Padova, Italy; serena.tedesco1988@gmail.com (S.T.); valentina.scattolini@gmail.com (V.S.); mattia.albiero@gmail.com (M.A.); angelo.avogaro@unipd.it (A.A.); andrea.cignarella@unipd.it (A.C.); 2 Veneto Institute of Molecular Medicine, Via G. Orus 2, 35129 Padova, Italy; mario.bortolozzi@unipd.it; 3 Department of Physics and Astronomy, University of Padova, Via Marzolo 8, 35131 Padova, Italy

**Keywords:** macrophage, phagocytosis, innate immunity, second messenger, calcium, mitochondria

## Abstract

Macrophages are highly plastic and dynamic cells that exert much of their function through phagocytosis. Phagocytosis depends on a coordinated, finely tuned, and compartmentalized regulation of calcium concentrations. We examined the role of mitochondrial calcium uptake and mitochondrial calcium uniporter (MCU) in macrophage polarization and function. In primary cultures of human monocyte-derived macrophages, calcium uptake in mitochondria was instrumental for alternative (M2) macrophage polarization. Mitochondrial calcium uniporter inhibition with KB-R7943 or MCU knockdown, which prevented mitochondrial calcium uptake, reduced M2 polarization, while not affecting classical (M1) polarization. Challenging macrophages with *E. coli* fragments induced spikes of mitochondrial calcium concentrations, which were prevented by MCU inhibition or silencing. In addition, mitochondria remodelled in M2 macrophages during phagocytosis, especially close to sites of *E. coli* internalization. Remarkably, inhibition or knockdown of MCU significantly reduced the phagocytic capacity of M2 macrophages. KB-R7943, which also inhibits the membrane sodium/calcium exchanger and Complex I, reduced mitochondria energization and cellular ATP levels, but such effects were not observed with MCU silencing. Therefore, phagocytosis inhibition by MCU knockdown depended on the impaired mitochondrial calcium buffering rather than changes in mitochondrial and cellular energy status. These data uncover a new role for MCU in alternative macrophage polarization and phagocytic activity.

## 1. Introduction

One of the most important biological functions of macrophages is phagocytosis. By removing micro-organisms, apoptotic bodies, and foreign particles, macrophages phagocytosis is instrumental to several physiological and pathological processes. An impairment in the phagocytic capacity of macrophages favours the spreading of infections [1] and contributes to tissue remodelling in airway disease [2], delayed wound healing [3], atherosclerosis [4,5], and myocardial infarction [6]. Vice versa, excess or frustrated phagocytosis can serve as a refuge for microbes or sustain inflammation, as in gout attacks [7]. Therefore, deciphering the molecular machinery of phagocytosis can reveal new attractive targets.

Calcium ions (Ca^2+^) are fundamental second messengers in phagocytosis. Imbalances in phagocyte calcium homeostasis have been linked to susceptibility to infection in diabetes [8] and chronic kidney disease [9]. An elevation of cytosolic calcium concentration ([Ca^2+^]c) is required for efficient phagocytosis and maturation of the phagosome [10]. Binding to phagocytic receptors on macrophages triggers the production of calcium-mobilizing second messengers, such as 1,4,5-inositol-triphosphate and sphingosine-1-phosphate. The ensuing calcium release from the endoplasmic reticulum leads to store-operated calcium entry from the extracellular space. Mobilization of calcium from lysosomes can also contribute to spikes of [Ca^2+^], but the role of mitochondrial calcium uptake in phagocytosis is largely unknown. The mitochondrial calcium uniporter (MCU) is a channel-forming 40 kDa protein integral to the mitochondrial inner membrane that allows calcium entry into the matrix [11]. The MCUb is a paralog and antagonist of MCU [12]. Although MCU is a major driver of mitochondrial calcium uptake, it has low affinity for calcium such that a high [Ca^2+^]c is required for a significant calcium entry into mitochondria. Nonetheless, mitochondria could uptake calcium when [Ca^2+^]c elevates, such as during phagocytosis, especially at microdomains of very high [Ca^2+^]c. Mitochondrial calcium uptake not only contributes to buffering [Ca^2+^]c, but also regulates mitochondrial function to determine cellular metabolism and cell death processes.

Macrophages are highly plastic cells that, upon trigger by extracellular stimuli, can acquire different activation states. Influx of extracellular calcium has been shown to be required for macrophage polarization toward the pro-inflammatory M1 phenotype [13], whereas lowering [Ca^2+^]c promoted an anti-inflammatory M2 switch [14]. In turn, M1 and M2 macrophages activate distinct pathways of internalization and signalling and differ in the processes of phagosome maturation [15]. M2 macrophages primed by IL-4/IL-13 were shown to be provided with higher phagocytic capacity than M1 macrophages primed by LPS/IFN-γ [16]. The role of MCU in these processes is less appreciated. In a previous study performed in mouse alveolar macrophages, MCU was found to be required for generation of pro-fibrotic macrophages, which share M2 features [17], by shifting cellular metabolism to fatty acid oxidation [18].

Based on these premises, we herein evaluated whether mitochondrial calcium uptake affected polarization of primary human monocyte-derived macrophages and their phagocytic activity.

## 2. Results

### 2.1. Mitochondrial Calcium Uptake Modulated Macrophage Polarization

**Figure 1:**
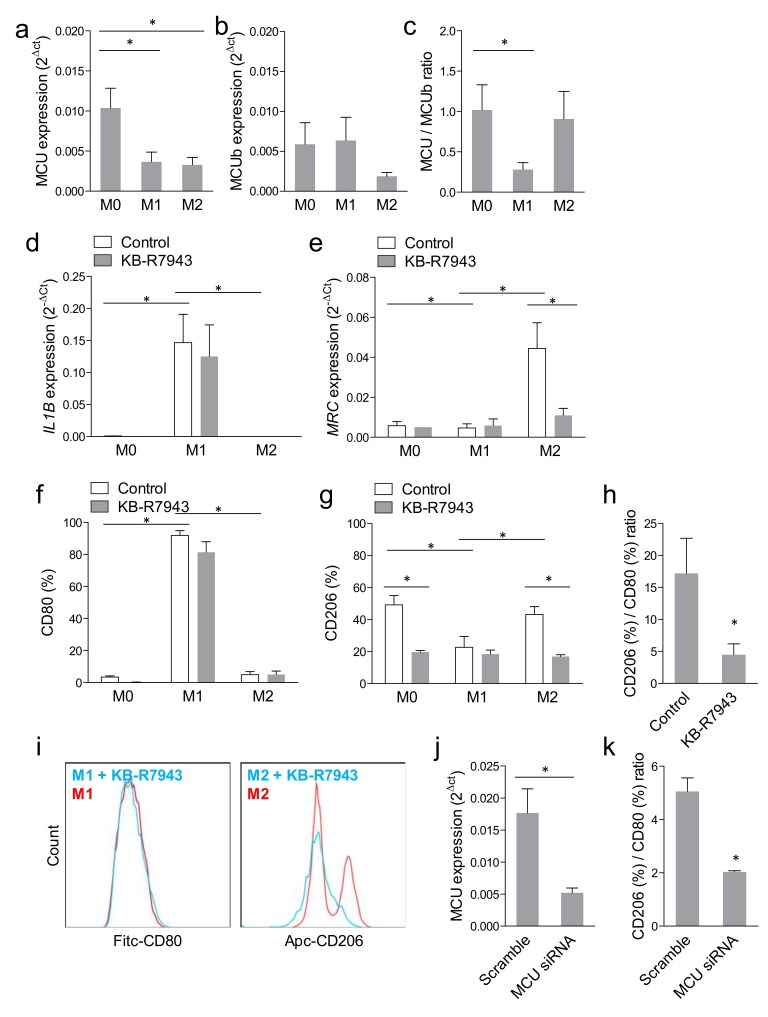
Mitochondrial calcium uniporter and macrophage polarization. Gene expression of *MCU* (**a**) and *MCUb* (**b**) was examined in M0 (resting), M1- and M2-polarized macrophages and the ratio between expression of MCU and MCUb (**c**) was calculated (* *p* < 0.05 for the indicated comparison after ANOVA). Gene expression of The M1 marker IL1B (**d**) and of the M2 marker *MRC* (**e**) was examined in M0, M1, and M2 macrophage with or without treatment with the MCU inhibitor KB-R7943 (* *p* < 0.05 for the indicated comparison after ANOVA). Surface expression of the M1 marker CD80 (**f**) and of the M2 marker CD206 (**g**) was examined by flow cytometry in M0, M1, and M2 macrophages with or without treatment with the MCU inhibitor KB-R7943 (* *p* < 0.05 for the indicated comparison after ANOVA). (**h**) The ratio of the surface expression of CD80 over CD206, which is considered a proxy of M1 polarization, was examined in M2-polarized cells, with or without treatment with the MCU inhibitor KB-R7943 (* *p* < 0.05). (**i**) Representative FACS plots of CD80 surface expression in M1 (left) and CD206 expression in M2 (right) macrophages with or without treatment with the MCU inhibitor KB-R7943. (**j**) Efficiency of siRNA-mediated knockdown of *MCU* in cells transfected with scramble siRNA or siRNA against *MCU* (* *p* < 0.05). (**k**) The ratio of the surface expression of CD206 over CD80 was examined in M2-polarized cells, with or without MCU knockdown (* *p* < 0.05).

Gene expression of *MCU* and *MCUb* was analysed in resting (M0), LPS/IFN-y-stimulated pro-inflammatory (M1) and IL-4/IL-13-stimulated anti-inflammatory (M2) macrophages. We observed a significant downregulation of *MCU* in M1 and M2 versus M0 (Figure 1a) and a trend lower *MCUb* gene expression in M2 versus M1 (Figure 1b). As a result, M1 macrophages displayed a significantly reduced *MCU*/*MCUb* gene expression ratio (Figure 1c), which is expected to result in a blunted mitochondrial Ca^2+^ uptake [19].

To evaluate whether mitochondrial calcium uptake affected polarization of macrophages, we blocked MCU with the chemical inhibitor KB-R7943. To confirm that KB-R7943 effectively inhibited mitochondrial calcium uptake, we imaged resting macrophages pre-loaded with Rhod-2 and Fluo-4 with or without KB-R7943 pre-treatment, during stimulation with ionomycin. To verify the mitochondrial specificity of Rhod-2 versus the cytoplasmic Ca^2+^ green dye Fluo-4, we performed co-localization experiments of Rhod-2 or Fluo-4 with Mitotracker green or red, respectively. According to the correlation plot analysis, Rhod-2, but not Fluo-4, co-localized with Mitotracker, thereby confirming mitochondrial specificity (Figure S1).

While [Ca^2+^]m markedly increased as a consequence of [Ca^2+^]c increase after ionomycin in control macrophages (Figure S2a), the surge in [Ca^2+^]m was significantly reduced by KB-R7943-treated macrophages (Figure S2b). Indeed, the ratio of Rhod-2 vs Fluo-4 signals was significantly lower in KB-R7943-treated macrophages (Figure S2c). This effect might not be explained by inhibition of Na^+^/Ca^2+^ exchanger (NCX) [20], either direct mode, which should increase both [Ca^2+^]c and [Ca^2+^]m, or reverse mode, which operates only after dissipation of the Na+ electrochemical gradient [21].

The KB-R7943 did not affect the induction of *IL1B* gene expression in M1-polarized cells (Figure 1d), but almost completely abolished the induction of *MRC* (encoding CD206) in M2-polarized cells (Figure 1e). Similarly, when evaluated by flow cytometry, KB-R7943 significantly reduced surface expression of the M2 marker CD206 in M0 and during the M2 polarization, without affecting surface expression of the M1 marker CD80 (Figure 1f,g). As a result, the ratio of CD206 over CD80 expression, which has been used as a summary statistics of human macrophage polarization [22,23], was significantly reduced by KB-R7943 to about one-third of the untreated control cells (Figure 1h,i). Since KB-R7943 can have off-target effects by inhibiting also NCX, we wished to confirm that modulation of MCU expression with siRNA affected macrophage polarization. Transfection with siRNA against *MCU* reduced *MCU* gene expression by >70% compared to macrophages transfected with scramble RNA (Figure 1j) and reduced MCU protein expression by >90% (Figure S3). Similar to was what observed with KB-R7943, *MCU* silencing reduced mitochondrial Ca^2+^ buffering occurring during rise in [Ca^2+^]c induced by ionomycin (Figure S2d–f). The MCU-silenced macrophages exposed to M2-polarizing stimuli showed a >2.5-fold reduction in the CD206/CD80 polarization index (Figure 1k).

Altogether, these data suggest that blocking mitochondrial calcium uptake by KB-R7943 or by knocking down MCU-blunted macrophage polarization towards M2. Next, we hypothesized that mitochondrial Ca^2+^ uptake is instrumental to one typical feature of M2, namely, phagocytosis.

### 2.2. Phagocytosis-Induced Mitochondrial Remodelling and Calcium Uptake

**Figure 2:**
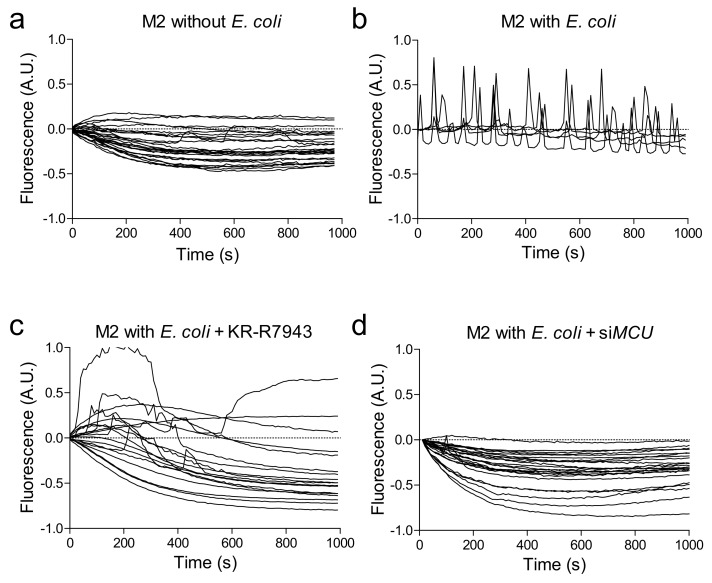
Mitochondrial calcium spikes during phagocytosis. Mitochondrial calcium concentrations, recorded as Rhod-2 fluorescence intensity, were imaged real-time in M2-polarized macrophages in the unstimulated condition (**a**) and after challenge with *E. coli* fragments without (**b**) or with (**c**) the MCU inhibitor KB-R7943 or transfection with siRNA against MCU (**d**). In each plot, individual lines represent the fluorescence intensity of regions of interest (ROI) drawn around each macrophage.

Macrophages were exposed in vitro to green fluorescent *Escherichia coli* fragments to stimulate phagocytosis. Live confocal multicolour imaging showed that contact of bacterial fragments elicited spikes of [Ca^2+^]m in 49% of macrophages pre-loaded with the mitochondrial calcium red dye Rhod-2 (Figure 2a,b and Supplementary Materials Video S1).

**Figure 3:**
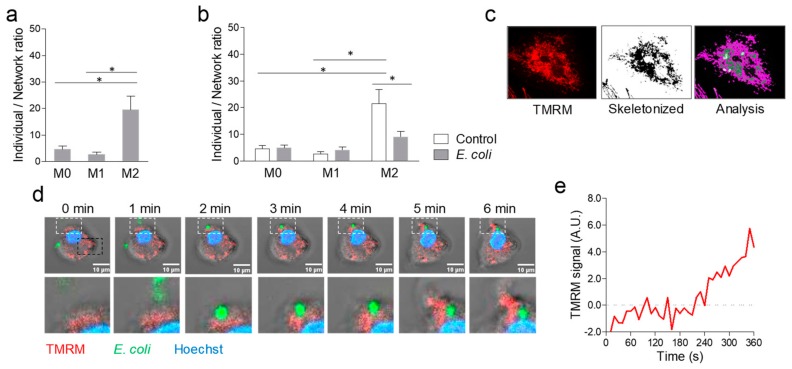
Mitochondrial remodelling during phagocytosis. Using the mitochondrial network analysis (MiNA) tool on macrophages stained with TOMM20, we calculated the ratio between individual macrophages and a macrophage network. (**a**) Average individual/network ratio in resting (M0), M1-, and M2-polarized macrophages (* *p* < 0.05 after ANOVA). (**b**) Average individual/network ratio in resting (M0), M1-, and M2-polarized macrophages before and after challenge with *E. coli* fragments (* *p* < 0.05 after 2-way ANOVA). (**c**) Representative images of a region of interest (ROI, ~40 µ) set around one macrophage to show the pattern of TOMM20 staining, result of the skeletonization procedure after imaging processing, and the analysis with MiNA tool. (**d**) Time-series obtained during live recording, cropped around a single M2 macrophage ROI (~40 µ), to show internalization of an *E. coli* fragment. Macrophages were stained with Hoechst 33342 and TMRM. The lower lane of images represents magnifications of the white, dashed insert of images in the upper lane. (**e**) TMRM fluorescence intensity over time in the white, dashed insert, where *E. coli* fragment internalization occurred, after subtraction of the TMRM fluorescence intensity in an equal remote ROI (black, dashed line).

It has been previously shown that phagocytosis of large apoptotic cells (ACs) results in remodelling of mitochondria, especially close to phagosomes. Since *E. coli* fragments are considerably smaller, we wished to verify such finding and used the mitochondrial network analysis (MiNA) tool on macrophages in resting condition (M0) or polarized toward M1 and M2 and stained with TOMM20 with or without challenging with *E. coli*. In unstimulated conditions, M2 macrophages showed a higher individual/network ratio (Figure 3a), suggestive of the presence of more fragmented mitochondria, than in M1 and M0 macrophages. After challenging with *E. coli*, the ratio did not change in M0 and M1 macrophages, but declined significantly in M2 macrophages, which was suggestive of fusion, rather than fission (Figure 3b,c). Therefore, contrary to what was observed during phagosome formation around large engulfed AC, phagocytosis of small bacterial fragments did not appear to induce mitochondrial fission. Nonetheless, using live imaging, we found that mitochondria rearranged close to sites of *E. coli* phagocytosis (Figure 3d), with an increase in TMRM signals close to the internalized particle relative to a remote cell area (Figure 3e), was more suggestive of localized fusion, rather than fission.

### 2.3. Blocking MCU Inhibited Macrophage Phagocytosis

To evaluate whether mitochondrial calcium uptake had any role in phagocytosis, we blocked MCU with the chemical inhibitor KB-R7943. When M2 macrophages pre-loaded with Rhod-2 and treated with KB-R7943 were challenged with *E. coli*, the vast majority of cells (95%) did not show any [Ca^2+^]m spike and, in a small number of cells, spike frequency was much lower and duration longer than in untreated cells (Figure 2c and Supplementary Materials Video S2).

**Figure 4:**
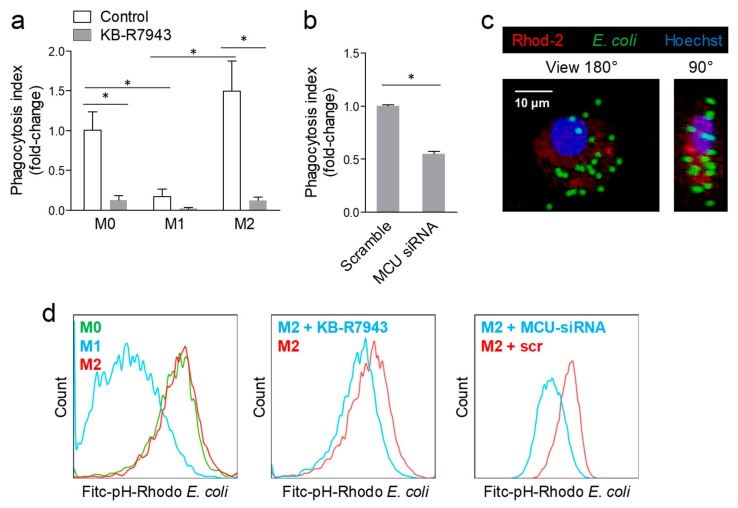
Blocking MCU inhibited phagocytosis. (**a**) Phagocytosis of *E. coli* fragments was quantified using flow cytometry in resting (M0), M1- and M2-polarized macrophages in the control condition or after pre-treatment with the MCU inhibitor KB-R7943 (* *p* < 0.05 after 2-way ANOVA). (**b**) Phagocytosis of *E. coli* fragments was quantified in M2-polarized macrophages transfected with scramble siRNA, or siRNA against MCU (* *p* < 0.05). (**c**) Representative 3D reconstruction of z-stacks collected during live imaging of M2 macrophages with confocal multiphoton multicolour microscopy. Cells were stained with the mitochondrial red calcium dye Rhod-2, the nuclear dye Hoechst 33342, while the green fluorescence derives from *E. coli* fragments (pH-Rhodo). The 180° and 90° rotated images are shown. (**d**) Representative FACS histograms showing phagocytic capacity of resting (M0), M1- and M2-polarized macrophages, as well as the effects of the MCU inhibitor KB-R7943 or *MCU* silencing on phagocytosis by M2 macrophages. The histogram shows, for each condition, the fluorescence intensity of the green (Fitc) channel on the *x*-axis, relative to the normalized cell count on the *y*-axis.

As expected, macrophages polarized to M1 displayed a significantly lower phagocytosis capacity compared to resting (M0) and M2 (Figure 4a), as determined by flow cytometry. In all cases, MCU inhibition with KB-R7943 markedly reduced phagocytosis (Figure 4a): by 87%, in M0, by 90% in M1 (non-significant due to low baseline phagocytosis rate), and by 92% in M2. In order to avoid confounding by off-target effects of KB-R7943, we knocked down *MCU* in resting macrophages by siRNA. Compared to scramble-transfected cells, cells treated with *MCU*-siRNA and challenged with *E. coli* displayed no [Ca^2+^]m spikes (Figure 2d). Finally, *MCU* silencing, compared with scramble siRNA transfection, resulted in a 50% reduction in phagocytosis capacity (Figure 4b–d).

**Figure 5:**
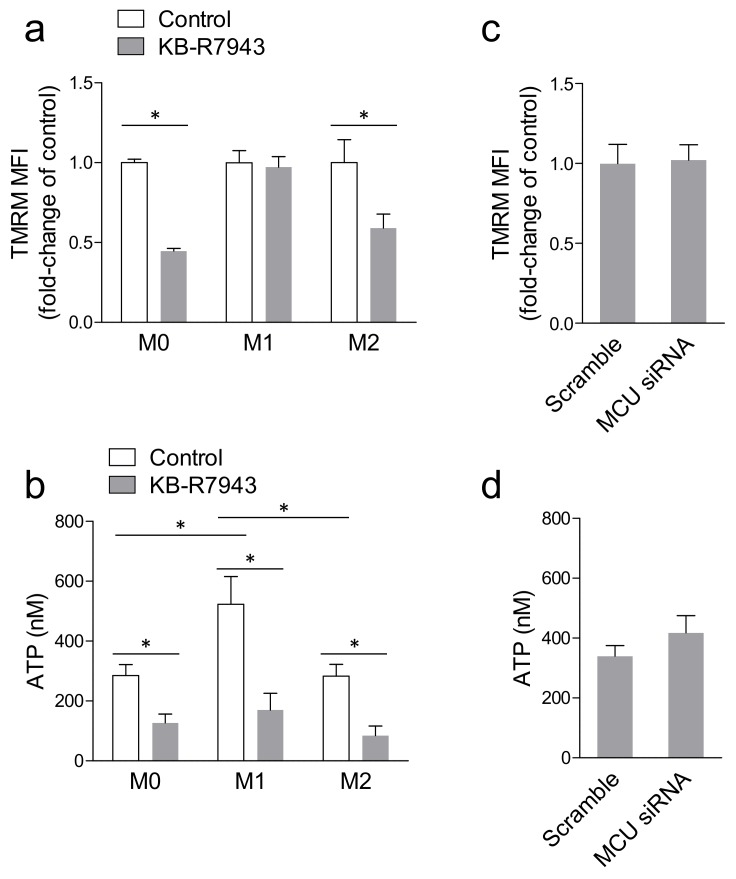
Effects of MCU blocking on mitochondrial potential and ATP levels. (**a**) Mitochondrial membrane potential was evaluated by flow cytometry in resting (M0), M1- and M2-polarized macrophages loaded with TMRM, with and without the MCU inhibitor KB-R7943: * *p* < 0.05 for the comparison between control (set at 1.0) and KB-R7943. (**b**) Cellular ATP levels were measured by a luminescence assay in M0, M1, and M2 macrophages with and without the MCU inhibitor KB-R7943 (* *p* < 0.05 after 2-way ANOVA). (**c**) Average TMRM fluorescence intensity in M2 macrophages transfected with scramble siRNA or with siRNA against MCU. (**d**) Cellular ATP levels in M2 macrophages transfected with scramble siRNA (set al. 1.0) or with siRNA against *MCU*.

The chemical MCU inhibitor KB-R7943 reduced macrophage mitochondrial Δψ as evidenced by the significantly lower TMRM intensity in M0 and M2 cells (Figure 5a), which have more prominent phagocytic activity compared to M1 (Figure 4a). In addition, cellular ATP levels, which were significantly higher in M1 versus M0 and M2, were reduced by KB-R7943 in all conditions (Figure 5b). Using *MCU*-siRNA in M2 macrophages, we found that neither TMRM signal (Figure 5c) nor cellular ATP levels (Figure 5d) were affected, thereby ruling out that modulation of phagocytosis depended on mitochondrial depolarization or impaired energy production.

## 3. Discussion

In this study, we evaluated the role of mitochondrial calcium uptake in determining macrophage polarization and function. Our data suggest that change in MCU expression is instrumental to M2 macrophage polarization and phagocytic activity. Briefly, we found that gene expression of MCU’s complex components changed according to the macrophage polarization status, suggestive of an overall reduced mitochondrial calcium uptake in M1. Blocking MCU reduced features of M2 polarization, evidenced by gene and protein expression of the typical scavenger receptor CD206. These data are in line with recent observations that MCU affects metabolism of alveolar mouse macrophage, shifting to a pro-fibrotic M2-like phenotype [17,18]. In turn, we found that M2 macrophages displayed distinctive phagocytic activity, which could be blunted by pharmacologic inhibition or genetic downregulation of MCU. Thus, we herein discovered a novel molecular program that could be used to modulate phagocytosis by human macrophage.

We wish to underline that most key findings we obtained with the chemical inhibitor KB-R7943 were confirmed in *MCU*-silenced cells. This is important because KB-R7943 is also a well-known inhibitor of NCX [20]. Although NCX isoforms are widely expressed [24], in our experimental setting, we found no evidence that KB-R7943 increased [Ca^2+^]c, as it would be predicted by NCX inhibition under normal Na^+^ gradients. Also, the literature provides no evidence for a role of NCX in macrophage phagocytic activity. Rather, we clearly found that KB-R7943 inhibited mitochondrial calcium buffering during [Ca^2+^]c overload and induction of [Ca^2+^]m spikes during phagocytosis. Similar effects on [Ca^2+^]m were observed in *MCU*-silenced macrophages. Importantly, *MCU* silencing modified features of M2 polarization and phagocytosis. In spite of these similarities, striking differences were noted when comparing the effects of KB-R7943 and *MCU* silencing on mitochondrial energization and cellular ATP levels. Reduction of TMRM signal and of ATP levels in cells treated with KB-R7943, but not in *MCU*-silenced cells, is consistent with the observation that KB-R7943 may also inhibit complex I [25]. This explains the stronger phagocytosis inhibition obtained with KB-R7943 (about 90%) than with *MCU* silencing (about 50%). Therefore, these data suggest that modulation of phagocytosis by *MCU* knockdown is unrelated to mitochondrial energization and ATP levels. The exact mechanism whereby mitochondrial calcium uptake potentiates phagocytosis is presently unclear. It should be noted that our experimental setting was only suitable to evaluate the early phases of phagocytosis, leading to internalization of *E. coli* fragments, whereas we did not assay later stages of phagosome maturation and processing. Phagosome formation is critically dependent on localized and coordinated regulation of calcium concentrations, instrumental to cytoskeletal dynamics and membrane fusion [10]. Therefore, we hypothesize that interfering with mitochondrial calcium buffering is sufficient to disrupt such delicate processes.

Our finding that MCU is required for phagocytosis by macrophages are apparently in contrast with a recent report that phagocytosis of ACs promoted mitochondrial fission via Drp1, thereby blunting buffering of reticulum-derived calcium in mitochondria, allowing an increase in [Ca^2+^]c which was required for phagolysosome formation and maturation [26]. The *MCU* silencing counteracted the defective AC phagocytosis induced by *Drp1* deletion, suggesting that excess calcium buffering by MCU in elongated mitochondria limited the surge in [Ca^2+^]c induced by ACs in macrophages [26]. To explain these contrasting findings, we first wish to underline that we always used primary cultures of human monocyte-derived macrophages, which can have different behaviour compared with bone-marrow-derived murine macrophages. Second, we used *E. coli* fragments, which are considerably smaller than AC, and their phagocytosis has likely lower requirements in terms of energetics, cytoskeletal rearrangement, and membrane dynamics. Finally, *Drp1* deletion may have generated physiologic deviations in cell function wherein MCU might well exert opposing effects on phagocytosis than in the wild type condition.

Our study has limitations. First, we used a small number of M1 and M2 markers to evaluate polarization, although they are well validated. Second, we did not uncover the exact mechanism mediating the effects of MCU on phagocytosis. In addition, the use of human primary macrophage culture forced us to employ non-ratiometric calcium dyes, somehow limiting the reliability of quantitative calcium imaging. Further, although MCU silencing achieved 70%−90% knockdown, residual MCU activity could have interfered with results. Finally, we have no data on whether similar effects would be observed for the phagocytosis of other biological particles or Gram-positive bacteria.

In summary, we show that mitochondrial calcium uptake is required for alternative polarization of human macrophages and for their phagocytic activity. While the role for MCU in modulating macrophage phenotype is being elucidated by others [17,18], our study shows for the first time that MCU regulates phagocytosis. These findings can have implications for our understanding of macrophage physiology and for the development of new therapeutics in conditions of insufficient or frustrated phagocytosis.

## 4. Materials and Methods

### 4.1. Differentiation and Polarization of Human Monocyte-Derived Macrophages

Blood was obtained from anonymized male, non-smoking healthy donors aged 18-35, at the blood collection clinic of the University Hospital of Padua, following institutional standard operating procedures. Samples were collected within the Biobank of the Department of Medicine (approved by the IRB of the University Hospital of Padova on 11/12/2006) and covered by project number 2786P. The PBMCs from buffy coats were isolated first by Ficoll–Paque (GE Healthcare, Chicago, IL, USA) density gradient centrifugation at 600 *g* for 30 min followed by a second high-density hyperosmotic Percoll gradient (GE Healthcare) at 600 *g* for 15 min. Monocytes were seeded at 5 × 10^5^/mL in RPMI 1640 medium supplemented with 10% FBS (Invitrogen, Carlsbad, CA, USA), 2 mM l-glutamine, 100 U/mL penicillin, and 100 μg/mL streptomycin in the presence of 20 nM CSF-1 [27]. Cells were cultured for 7 days at 37 °C and 5% CO_2_, with medium change every 3 days, to obtain human monocyte-derived macrophages (hMDM). After removing the culture medium, resting macrophages were polarized toward M1 phenotype by incubation for 48 h with LPS (1 μg/mL, Sigma-Aldrich, St. Luis, MO, USA) and IFN-γ (10 ng/mL). The M2 polarization was obtained by adding IL-4 (20 ng/mL) and IL-13 (5 ng/mL; all Immunotools, Friesoyte, Germany) for 48 h, as previously described [28]. At the end of the activation protocol, where indicated, cells were incubated for 30 min with 10 nM KB-R7943 mesylate (Tocris, Bristol, UK).

### 4.2. Gene Expression Analysis

Total RNA was isolated from 3 × 10^5^ cells. Cells were harvested, washed once in PBS, and RNA was extracted using the Total RNA Purification Plus Kit (Norgen Bioteck Corporation, Schmon Pkwy, Thorold, CA). The cDNA was generated from 500 ng total RNA using SensiFAST cDNA Syntesis Kit (Bioline, Cincinnati, OH, USA) according to the manufacturer’s instructions. The relative quantification of the genes of interest was measured by real-time quantitative PCR (qPCR) performed using SensiFAST SYBR Hi-ROX Kit (Bioline, Cincinnati, OH, USA) with the following settings: 95 °C for 2 min (1 cycle), then 95 °C for 5 s, followed by 20 s at 60 °C (40 repeats). Oligonucleotide primers were designed using the online tool for Real-Time PCR Blast and obtained from Invitrogen. Results were normalized to Actin as housekeeping gene as reference and evaluated using the 2ΔΔCt method. The primer sequences were as follows: *hMCU*: FW GCAGAATTTGGGAGCTGTTT; RW GTCAATTCCCCGATCCTCTT. *hMCUb*: FW GGCCTTCCCTTGGTAACACT; RW GTTGCCATCTGCTGTGAAGA. *hMRC*: FW CCTCTGGTGAACGGAATGAT; RW AGGCCAGCACCCGTTAAAAT. *hIL1b*: FW CAGCCAATCTTCATTGCTCA; RW TCGGAGATTCGTAGGTGGAT. *hACTB*: FW GGATGCCACAGGACTCCA; RW AGAGCTACGAGCTGCCTGAC.

### 4.3. Flow Cytometry

Surface antigenic expression of M0-, M1-, and M2-polarized hMDMs was analysed by flow cytometry. Adherent cells were washed once with PBS, gently scraped and transferred into FACS tubes (BD Biosciences Pharmigen, San Diego, CA, USA). Cells were then stained with fluorochrome-tagged monoclonal antibodies (all from BD Biosciences) against surface CD80 (FITC) as M1 macrophage marker, and against CD206 (APC) as an M2 marker. This panel of M1/M2 markers was based on previous characterizations [28,29,30]. Samples were washed, suspended in PBS, and 30,000 events for each sample were recorded in a FacsCanto II flow cytometer (BD Biosciences). For quantitation of the TMRM signal, cells were collected as described before and incubated in the dark for 30 min in the presence of 20 nM TMRM. Cells then were washed and analysed with FacsCanto II flow cytometer. Data were analysed using the FlowJo software (10.4.2, FlowJo LLC, Ashland, OR, USA).

### 4.4. Western Blot

Cells were lysed with RIPA lysis buffer. After centrifugation at 10,000× g for 15 min, supernatants were harvested for SDS-PAGE and Western blotting. Protein quantification was performed by the BCA assay (Sigma). Proteins (40 μg) were separated on SDS-PAGE and transferred onto PVDF membranes. Membranes were then blocked and probed using the following primary antibodies: rabbit anti-MCU (1:1000; Cell Signaling, Danvers, MA, USA) and mouse anti-GAPDH at 1:5000 (Santa Cruz, Santa Cruz, CA, USA). After washing, membranes were incubated with appropriate secondary HRP-conjugated antibodies (Jackson ImmunoResearch, Bar Harbor, ME, USA) at 1:10,000. Bands were detected by chemiluminescence using the LiteAblot Turbo (Euroclone, Milan, Italy). Images were acquired with Image Quant Las 4000 (GE Healthcare, Chicago, IL, USA). Densitometric analysis was performed with Image J 1.47v (NIH, USA).

### 4.5. Phagocytosis Assays

Phagocytosis assay was performed in resting (M0), M1- and M2-polarized hMDMs treated with KB-R7943 or after MCU knockdown. After 48 h polarization or silencing, cells were incubated with fluorescent PhrodoTM Green *E. coli* bioparticles conjugate (ThermoFisher Scientific, Milan, Italy) for 1 h in serum-free RPMI at 37 °C, 5% CO_2_ [31]. Cells were then washed three times with cold PBS to remove fluorescent beads that had not been internalised. Finally, macrophages were scraped from the plate and analysed by flow cytometry or visualized by confocal microscopy.

### 4.6. ATP Luminescence Assay

The ATP levels were measured in activated macrophages in the presence or absence of MCU inhibitor, KB-R7943, and after siRNA-mediated MCU silencing. To perform a direct comparison, cells were harvested and counted, and the same number of cells were re-suspended in 100 µL. The ATPlite assay was performed according to the manufacturer’s instructions (PerkinElmer, Waltham, MA, USA). Luminescence was measured with a FLUOstar OPTIMA luminescence plate reader (BMG Labtech, Ortenberg, Germany).

### 4.7. Gene Silencing

Scramble siRNA control and oligo-targeting siRNA were transfected into macrophages using Lipofectamine 2000 (Invitrogen) at 20 nM siRNA in 24-well plates. Macrophages were incubated for 6 h in RMPI 1640 containing 3 μL Lipofectamine 2000 per 2 × 10^5^ cells and 75 pM siRNA mix [32]. At the end, the mix was removed and replaced with RMPI 1640 medium supplemented with 10% FBS and 10 nM CSF-1 with no antibiotics. Experiments and RNA analysis were conducted 48 hrs later. siRNA sequences were as follows: *hMCU* siRNA1 5′-GCCAGAGACAGACAAUACUtt-3′; 3′-ttCGGUCUCUGUCUGUUAUGA-5′; siRNA-2 5′-GGGAAUUGACAGAGUUGCUtt-3′; 3′-ttCCCUUAACUGUCUCAACGA-5′. *sMCUb* siRNA-1 5′-AUACUACCAGUCACACCAUtt-3′: 3′-ttUAUGAUGGUCAGUGUGGU-5′; siRNA-2 5′-UUUCUUCAGUUCUUCCACAtt-3′; 3′-ttAAAGAAGUCAAGAAGGUGU-5′. Scramble: 5′-GCCUAAGAACGACAAAUCAtt-3′; 3′-ttCGGAUUCUUGCUGUUUAGU-5′

### 4.8. Imaging Experiments

Dye loading. Culture medium was replaced by RMPI 1640 0% FBS and hMDM were loaded with 300 nM Mito-Tracker Green FM (Invitrogen) for 20 min, 1 mg/mL Hoechst 33342 (Sigma–Aldrich) for 5 min; calcium dyes, 5 μM Rhod-2 AM and 5 μM Fluo-4 AM (ThermoFisher), were loaded in the presence of 0.1% *w*/*v* Pluronic F–127 (Sigma–Aldrich) for 20 min and de-esterified for other 20 min in a CO_2_-incubator at 37°C.

Multi-photon setup. Cells are placed on a custom-made holder and positioned under a 25× objective optimized for multiphoton imaging (Olympus XLPLN25XWMP2, N.A. 1.05, W.D. 2 mm). In all live imaging experiments, we used a modular multiphoton microscope (Bergamo II, Thorlabs) equipped with a 8 kHz resonant scanner, pulsed laser beams generated by an optical parametric oscillator pumped by a Ti:Sapphire laser (Chameleon Ultra 2, Coherent), extended-field-of-view collection optics, 4 independent detection GaAsP photomultipliers (Hamamatsu) in the backward direction and a fast photodetector for laser scanning Dodt gradient-contrast imaging in the forward direction. Two-photon microscopy at 800 nm excitation was used to detect simultaneously calcium, mitochondrial and nuclear dyes and cell shape in transmitted light.

Mitochondrial dye co-localization. Cells double-loaded with 5 μM Rhod-2 AM and 300 nM Mito-Tracker Green, were simultaneously excited and emitted fluorescence was recorded in two separated channels. We monitored fluorescence intensity variations due to 10 μM ionomycin (Sigma) stimulus.

Phagocytosis. We monitored simultaneously the internalization of fluorescent beads (pHrodo™ Green *E. coli* BioParticles™ Conjugate for Phagocytosis, 1.5 μm diameter) and mitochondrial calcium activity using Rhod-2 dye, for 1 h in serum-free RPMI at 37°C. For co-localization analysis, we used an automatic algorithmic method to measure the amount of co-localization in two-colour, three-dimensional microscopic images [33]. This method works in Matlab environment (Release 14, The MathWorks, Inc., Natick, MA, USA) and first it measures the probability (*p*-value) that true colocalization is present in a selected region. We used a *p*-value > 95% to indicate true colocalization. As a second step, colocalized pixels in the selected region were identified using a statistical criterion based on the two-dimensional histogram of both channels allowing the computation of mitochondrial dyes being colocalized (white pixels).

Immunostaining. Polarized macrophages were fixed for 30 min in 4% paraformaldehyde at RT and permeabilized with 0.1 Triton X-100 for 10min. Cells were washed with PBS and stained with 1:200 anti-TOMM20 antibody (Abcam), for 1 h at RT. Secondary antibody staining was performed for 1 h with 1:200 Alexa-647, 1:60 Alexa Fluor™ 488 Phalloidin and 1mg/mL Hoechst 33342 (Sigma-Aldrich). Confocal images were collected with a Leica TCS SP5 using a 20× objective (HC PL APO CS 15506513, 0.70 NA, 0.59 mm WD). Optical sections were recorded at 250 nm per vertical step and four times averaging.

Mitochondrial network analysis. We analysed mitochondrial morphology through ImageJ macro tool, called Mitochondrial Network Analysis (MiNa). This ImageJ macro tool works using ImageJ existing plug-ins, pre-processing images making them filtered, binarized and skeletonized [34]. MiNA outputs give information about mitochondrial morphology (branches, punctuates, rods) and we evaluated mitochondrial fission/fusion during phagocytosis.

Calcium imaging. For calcium spikes analysis, we used an in-house developed analysis software, which works under Matlab environment and was designed to plot averaged time responses from specific regions of interest (ROIs) [35]. The fluorescence intensity was plotted as F/F0, where F0 is the pre-stimulus basal fluorescence. Statistical comparisons were made using the Mann–Whitney U test.

### 4.9. Statistical Analysis

Experiments were performed at least in triplicate using different donors. Several technical replicates were performed for each donor. Comparison among two or more groups were performed using Student’s *t*-tests or ANOVA, respectively. When two factors had to be analysed, two-way ANOVA was used. Statistical significance was set at *p* < 0.05.

## Supplementary Materials

Supplementary materials can be found at https://www.mdpi.com/1422-0067/20/19/4966/s1.

## References

[1] Thomas C.A., Li Y., Kodama T., Suzuki H., Silverstein S.C., El Khoury J. (2000). Protection from lethal gram-positive infection by macrophage scavenger receptor-dependent phagocytosis. J. Exp. Med..

[2] Donnelly L.E., Barnes P.J. (2012). Defective phagocytosis in airways disease. Chest.

[3] Sindrilaru A., Peters T., Schymeinsky J., Oreshkova T., Wang H., Gompf A., Mannella F., Wlaschek M., Sunderkotter C., Rudolph K.L. (2009). Wound healing defect of Vav3−/− mice due to impaired {beta}2-integrin-dependent macrophage phagocytosis of apoptotic neutrophils. Blood.

[4] Tabas I. (2010). Macrophage death and defective inflammation resolution in atherosclerosis. Nat. Rev. Immunol..

[5] Li S., Sun Y., Liang C.P., Thorp E.B., Han S., Jehle A.W., Saraswathi V., Pridgen B., Kanter J.E., Li R. (2009). Defective phagocytosis of apoptotic cells by macrophages in atherosclerotic lesions of *ob*/*ob* mice and reversal by a fish oil diet. Circ. Res..

[6] Bujak M., Kweon H.J., Chatila K., Li N., Taffet G., Frangogiannis N.G. (2008). Aging-related defects are associated with adverse cardiac remodeling in a mouse model of reperfused myocardial infarction. J. Am. Coll. Cardiol..

[7] Busso N., So A. (2010). Mechanisms of inflammation in gout. Arthritis Res. Ther..

[8] Alexiewicz J.M., Kumar D., Smogorzewski M., Klin M., Massry S.G. (1995). Polymorphonuclear leukocytes in non-insulin-dependent diabetes mellitus: Abnormalities in metabolism and function. Ann. Intern. Med..

[9] Krol E., Agueel R., Banue S., Smogorzewski M., Kumar D., Massry S.G. (2003). Amlodipine reverses the elevation in [Ca^2+^]i and the impairment of phagocytosis in PMNLs of NIDDM patients. Kidney Int..

[10] Nunes P., Demaurex N. (2010). The role of calcium signaling in phagocytosis. J. Leukoc. Biol..

[11] De Stefani D., Raffaello A., Teardo E., Szabo I., Rizzuto R. (2011). A forty-kilodalton protein of the inner membrane is the mitochondrial calcium uniporter. Nature.

[12] Marchi S., Pinton P. (2014). The mitochondrial calcium uniporter complex: Molecular components, structure and physiopathological implications. J. Physiol..

[13] Zhou X., Yang W., Li J. (2006). Ca^2+^- and protein kinase C-dependent signaling pathway for nuclear factor-kappaB activation, inducible nitric-oxide synthase expression, and tumor necrosis factor-alpha production in lipopolysaccharide-stimulated rat peritoneal macrophages. J. Biol. Chem..

[14] Kang H., Zhang K., Wong D.S.H., Han F., Li B., Bian L. (2018). Near-infrared light-controlled regulation of intracellular calcium to modulate macrophage polarization. Biomaterials.

[15] Canton J. (2014). Phagosome maturation in polarized macrophages. J. Leukoc. Biol..

[16] Tarique A.A., Logan J., Thomas E., Holt P.G., Sly P.D., Fantino E. (2015). Phenotypic, functional, and plasticity features of classical and alternatively activated human macrophages. Am. J. Respir. Cell. Mol. Biol..

[17] Gu L., Larson-Casey J.L., Carter A.B. (2017). Macrophages utilize the mitochondrial calcium uniporter for profibrotic polarization. FASEB J..

[18] Gu L., Larson Casey J.L., Andrabi S.A., Lee J.H., Meza-Perez S., Randall T.D., Carter A.B. (2019). Mitochondrial calcium uniporter regulates PGC-1alpha expression to mediate metabolic reprogramming in pulmonary fibrosis. Redox Biol..

[19] Raffaello A., De Stefani D., Sabbadin D., Teardo E., Merli G., Picard A., Checchetto V., Moro S., Szabo I., Rizzuto R. (2013). The mitochondrial calcium uniporter is a multimer that can include a dominant-negative pore-forming subunit. EMBO J..

[20] Watano T., Harada Y., Harada K., Nishimura N. (1999). Effect of Na^+^/Ca^2+^ exchange inhibitor, KB-R7943 on ouabain-induced arrhythmias in guinea-pigs. Br. J. Pharmacol..

[21] DiPolo R., Beauge L. (2006). Sodium/calcium exchanger: Influence of metabolic regulation on ion carrier interactions. Physiol. Rev..

[22] Tedesco S., Adorni M.P., Ronda N., Cappellari R., Mioni R., Barbot M., Pinelli S., Plebani M., Bolego C., Scaroni C. (2019). Activation profiles of monocyte-macrophages and HDL function in healthy women in relation to menstrual cycle and in polycystic ovary syndrome patients. Endocrine.

[23] Toniolo A., Fadini G.P., Tedesco S., Cappellari R., Vegeto E., Maggi A., Avogaro A., Bolego C., Cignarella A. (2015). Alternative activation of human macrophages is rescued by estrogen treatment in vitro and impaired by menopausal status. J. Clin. Endocrinol. Metab..

[24] Khananshvili D. (2014). Sodium-calcium exchangers (NCX): Molecular hallmarks underlying the tissue-specific and systemic functions. Pflugers Arch..

[25] Brustovetsky T., Brittain M.K., Sheets P.L., Cummins T.R., Pinelis V., Brustovetsky N. (2011). KB-R7943, an inhibitor of the reverse Na^+^/Ca^2+^ exchanger, blocks N-methyl-D-aspartate receptor and inhibits mitochondrial complex I. Br. J. Pharmacol..

[26] Wang Y., Subramanian M., Yurdagul A., Barbosa-Lorenzi V.C., Cai B., De Juan-Sanz J., Ryan T.A., Nomura M., Maxfield F.R., Tabas I. (2017). Mitochondrial Fission Promotes the Continued Clearance of Apoptotic Cells by Macrophages. Cell.

[27] Repnik U., Knezevic M., Jeras M. (2003). Simple and cost-effective isolation of monocytes from buffy coats. J. Immunol. Methods.

[28] Tedesco S., Bolego C., Toniolo A., Nassi A., Fadini G.P., Locati M., Cignarella A. (2015). Phenotypic activation and pharmacological outcomes of spontaneously differentiated human monocyte-derived macrophages. Immunobiology.

[29] Fadini G.P., De Kreutzenberg S.V., Boscaro E., Albiero M., Cappellari R., Krankel N., Landmesser U., Toniolo A., Bolego C., Cignarella A. (2013). An unbalanced monocyte polarisation in peripheral blood and bone marrow of patients with type 2 diabetes has an impact on microangiopathy. Diabetologia.

[30] Murray P.J., Allen J.E., Biswas S.K., Fisher E.A., Gilroy D.W., Goerdt S., Gordon S., Hamilton J.A., Ivashkiv L.B., Lawrence T. (2014). Macrophage activation and polarization: Nomenclature and experimental guidelines. Immunity.

[31] Schrijvers D.M., Martinet W., De Meyer G.R., Andries L., Herman A.G., Kockx M.M. (2004). Flow cytometric evaluation of a model for phagocytosis of cells undergoing apoptosis. J. Immunol. Methods.

[32] Troegeler A., Lastrucci C., Duval C., Tanne A., Cougoule C., Maridonneau-Parini I., Neyrolles O., Lugo-Villarino G. (2014). An efficient siRNA-mediated gene silencing in primary human monocytes, dendritic cells and macrophages. Immunol. Cell. Biol..

[33] Costes S.V., Daelemans D., Cho E.H., Dobbin Z., Pavlakis G., Lockett S. (2004). Automatic and quantitative measurement of protein-protein colocalization in live cells. Biophys J..

[34] Valente A.J., Maddalena L.A., Robb E.L., Moradi F., Stuart J.A. (2017). A simple ImageJ macro tool for analyzing mitochondrial network morphology in mammalian cell culture. Acta Histochem..

[35] Ciubotaru C.D., Bastianello S., Beltramello M., Pozzan T., Mammano F., Adlassnig K.-P., Bracale M. (2005). Multi-Modal Imaging of Cytosolic and Mitochondrial Ca^2+^.

