# Supplementary material for: Mitochondrial Calcium Uptake Is Instrumental to Alternative Macrophage Polarization and Phagocytic Activity
Source: Int J Mol Sci. 2019 Oct 8;20(19):4966. doi: 10.3390/ijms20194966 (PMC6801659; doi:10.3390/ijms20194966)
Supplement: Supplementary file 1 [file ijms-20-04966-s001.pdf]

International Journal of *Molecular Sciences*

MDPI

Article

Supplementary Materials:Mitochondrial Calcium Uptake is Instrumental to Alternative Macrophage Polarization and Phagocytic Activity

Serena Tedesco, Valentina Scattolini, Mattia Albiero, Mario Bortolozzi, Angelo Avogaro, Andrea Cignarella, Gian Paolo Fadini

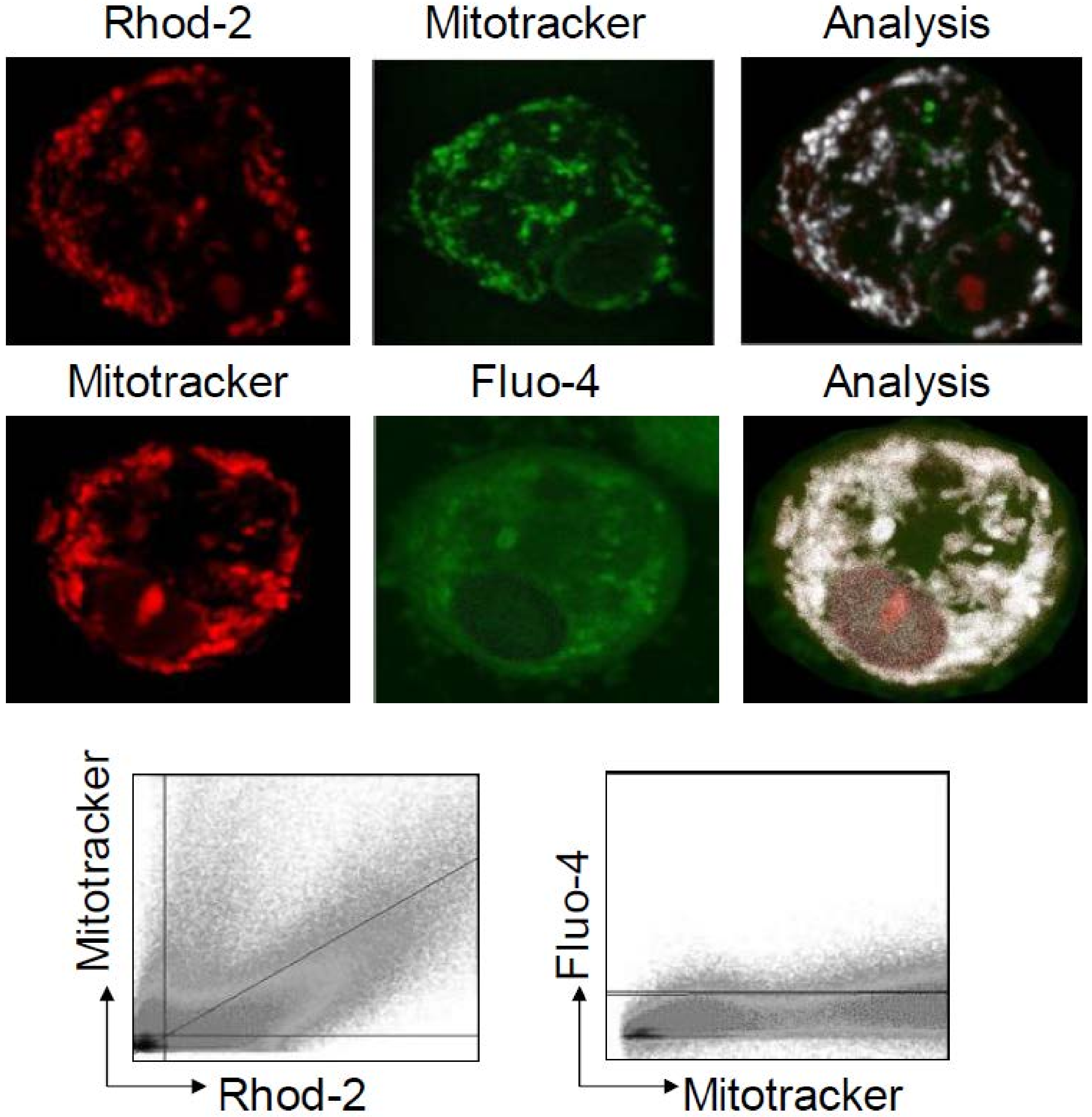

Figure 1. Mitochondrial localization of Rhod-2 and Fluo-4. Mitochondria were stained with Mitotracker red or green, and calcium was imaged with the green cytoplasmic dye Fluo-4 or the red mitochondrial dye Rhod-2, respectively. A correlation matrix was built, where the fluorescence intensity of each pixel in the same X/Y position of the microscopic image was plotted for the two channels considered. Thus, in case of 100% co-localization of two fluorescence channels, all pixels would lie on the same line. In case of no fluorescence co-localization, the coupled dots would be scattered without any linear arrangement. The white colour in the right panels identifies pixels in the image where green and red co-localized: co-localization is confirmed if the same pixel stains green,

red and white in the three images. As evident from the correlation matrix, Rhod-2 colocalized with Mitotracker green (dots were arranged around a correlation line) whereas Fluo-4 did not colocalize with Mitotracker red (no correlation trend was detected).

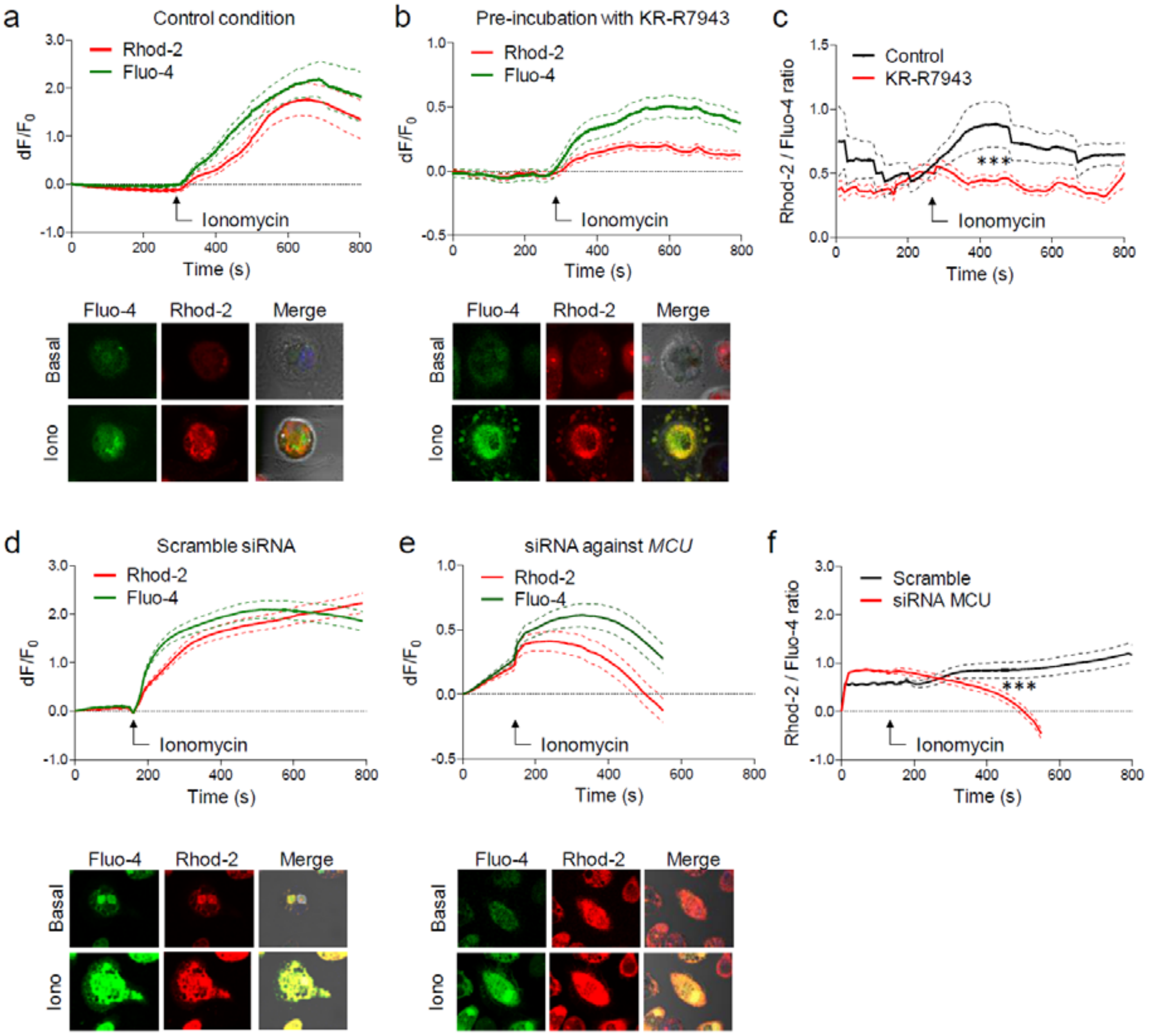

Figure 2. Calcium imaging. Using live multicolour multiphoton microscopy, we imaged the signal of the cytoplasmic green calcium dye Fluo-4 and of the mitochondrial red calcium dye Rhod-2 in resting macrophages, before and after stimulation with the calcium ionophore ionomycin, at about 300 s (arrow). At least 20 cells were imaged during each experiment, setting independent regions of interest (ROI). The green and red lines indicate average signals of all ROI, whereas dashed lines indicated 95% confidence interval (C.I.) of the mean. a) Cells imaged in the control condition. b) Cells were pre-treated with the MCU inhibitor KB-R7943. c) The mean ratio between Rhod-2 and Fluo-4 signals is reported over time, with 95% C.I. for the two conditions shown in (a) and (b). *** $p<0.001$. d) Cells transfected with scramble siRNA. e) Cells transfected with siRNA against MCU. f) The mean ratio between Rhod-2 and Fluo-4 signals is reported over time, with 95% C.I. for the two conditions shown in (d) and (e). *** $p<0.001$. Below graphs in (a), (b), (d) and (e) are representative ROI of the Fluo-4, Rhod-2 and nuclear (Hoechst 33342, blue) staining before and after stimulation with ionomycin (iono). Yellow/orange staining in the merged image panels indicate simultaneous surge in Rhod-2 and Fluo-4 fluorescence intensity. Absolute fluorescence values may not comparable among different experiments, due to changing focus and overall intensity of the staining. Having set at zero the initial fluorescence in both channels, the change over time and their ratio reflects the differences between the conditions being compared.

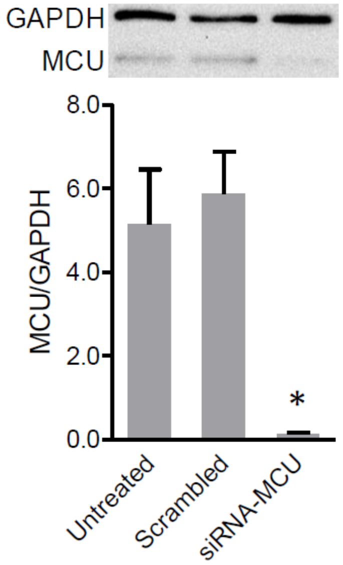

Figure 3. Western blot of MCU in control and silenced cells. Proteins extracted from untreated macrophages, macrophages transfected with scramble siRNA, and macrophage transfected with siRNA against MCU were loaded. Quantification was performed semiquantitatively from two replicates. *$p<0.05$ versus scrambled and versus control untreated cells.

Video 1 and 2. Spikes of mitochondrial calcium during phagocytosis. M2 macrophages were loaded with the mitochondrial calcium red dye Rhod-2 and imaged during challenge with green fluorescent *E. coli* fragments, using live confocal multiphoton microscopy. Video 1 shows that M2 macrophages undergo multiple spikes of Rhod-2 signal (represented graphically in Figure 4b). Video 2 shows that M2 macrophages pre-treated with the MCU inhibitor KB-R7943 displayed no Rhod-2 spike, despite challenge with *E. coli*. In both videos the cropped ROI is about ~40 m.

Submitted for possible open access publication under the terms and conditions of the Creative Commons Attribution (CC BY) license (http://creativecommons.org/licenses/by/4.0/).
